# Empathy at the Heart of Darkness: Empathy Deficits That Bind the Dark Triad and Those That Mediate Indirect Relational Aggression

**DOI:** 10.3389/fpsyt.2019.00095

**Published:** 2019-03-12

**Authors:** Nadja Heym, Jennifer Firth, Fraenze Kibowski, Alexander Sumich, Vincent Egan, Claire A. J. Bloxsom

**Affiliations:** ^1^Division of Psychology, Nottingham Trent University, Nottingham, United Kingdom; ^2^Department of Psychology, Auckland University of Technology, Auckland, New Zealand; ^3^Forensic and Family Psychology, University of Nottingham, Nottingham, United Kingdom

**Keywords:** dark triad, cognitive empathy, affective empathy, online simulation, relational aggression

## Abstract

The dark triad (DT) traits–psychopathy, narcissism and Machiavellianism–have collectively been linked to reduced empathy and increased aggression; however, their association with distinct empathic subtypes remains unclear; and unique links to indirect relational aggression (IRA) have not been delineated. Moreover, whether dark traits should be conceptualized individually, as a dyad or as a triad with a dark core centered around the absence of empathy is debated. The current study examines (i) whether impaired empathy indeed represents a common “dark core” binding Machiavellianism, narcissism, and psychopathy, and (ii) this core explains associations between the dark traits and IRA. Participants (*N* = 301, 262 F/39 M) completed measures of the DT traits, cognitive and affective empathy components and IRA (Social Exclusion, Malicious Humor and Guilt Induction). The individual traits model without links between narcissism and IRA showed the best fit, suggesting that, at least in the context of IRA, the DT traits are best viewed as three independent personality traits. Distinct cognitive and affective empathy deficits and capacities are seen in the DT. Peripheral responsivity was the only type of empathy deficit associated with all dark traits, but unrelated to IRA. Psychopathy was the strongest indicator of impaired empathy and all IRAs; however, only online simulation, an affect-related cognitive empathy facet, partially mediated the relationships of psychopathy and Machiavellianism with IRA. Whilst the unique pathways for the dark triad traits suggest stronger alignment of psychopathy and Machiavellianism in their empathic deficits and indirect aggression; the data do not support the notion that an unempathic dark core underpinning all three traits drives indirect aggression. This is the first paper delineating the specific empathic deficits involved using a facet approach and their link to indirect forms of aggression. Results therefore inform theoretical models of aggression in the DT and offer some clarity on the debates surrounding the unempathic dark core in the DT.

## Introduction

Empathy is central to prosocial interactions, understanding the suffering of others, and attenuates the propensity to maladaptive aggressive behavior (1). Reduced empathy, along with other traits, is proposed to represent a “core,” interconnecting three maladaptive personality constructs–Machiavellianism, narcissism, and psychopathy—which have been conceptualized as the Dark Triad [DT; (2, 3)], and associated with aggression (4). Whether this is indeed the case is fuelled by a lack of empirical findings that delineate facets of empathy underpinning the core and those more uniquely linked to the individual dark traits [e.g., (5)]. According to the Violence Inhibition Mechanism model [VIM; (6)], a selective impairment in the emotional recognition and response to distress cues, such as fearful and sad expressions, exacerbates aggression in psychopathy (7). However, less is known about the empathy-aggression link in the context of the other two DT traits. If aggression is driven by the absence of those empathic traits that also form the dark core, the VIM model would theoretically also apply to Narcissism and Machiavellianism. Conceptualizations of the dark traits as separate traits without an unempathic core, currently debated in the field [e.g., (8) for review; (9–13)], might challenge this notion. Moreover, whilst direct forms of physical aggression have been typically studied in this context, less is known about these associations for more indirect forms of aggression. The current study seeks to close this gap by examining (i) whether impaired empathy indeed represents a common “dark core” binding Machiavellianism, narcissism, and psychopathy, and (ii) whether empathy deficits explain associations between the dark traits and indirect relational aggression [IRA; (10)].

### In Search of the Core of Evil

Each DT trait has been described with unique characteristics: Machiavellianism describes an exploitative cynical nature, being a manipulator rather than manipulated (14, 15). Narcissism is characterized by an exaggerated sense of entitlement, superiority, and grandiose thinking (2). Psychopathy comprises a constellation of affective-interpersonal (superficial charm, callous affect) and behavioral (erratic lifestyle, antisocial behavior) deficits (16, 17). Nevertheless, the DT traits are significantly inter-correlated (18) with considerable convergent correlations between subscales (19).

Collectively, the DT traits share a propensity for a callous and manipulative interpersonal life-style, leading prior research to examine the empirical overlap between these subclinical personality traits, in order to designate the underlying core of “evil” personalities. The so-called “dark core” is proposed to comprise a set of traits and emotional deficits that is common across all three traits, explains their shared variance and promotes a selfish and antagonistic lifestyle (4). Most notably, callous-unemotional (CU) traits and empathic deficits have been proposed to constitute the shared dark core of all three dark traits (3), as well as low Agreeableness (2), low Honesty-Humility (13, 20, 21), and a behavioral overlap of an alternative fast and exploitative life history strategy (22).

Importantly, there are debates in the field as to how the DT should be best conceptualized–as individual traits or shared constructs with a joint dark core (8). For example, some authors propose that narcissism is less central to the dark core than psychopathy and Machiavellianism, and best viewed as a separate construct, leaving psychopathy and Machiavellianism as a dark dyad [e.g., (10, 23)]. Indeed, a factor analytic study showed a stronger clustering of psychopathy and Machiavellianism with other variables (e.g., moral disengagement, unethical attitudes, and disagreeableness) capturing antisocial variance, whereas narcissism was much stronger associated with a non-antisocial factor alongside traits such as extraversion and intellect (11). Psychopathy and Machiavellianism share more overlap than they do with narcissism, and greater similarity in their associations with other CU personality correlates [i.e., low Agreeableness and Conscientiousness; (12)]. Nevertheless, confirmatory factor analyses suggest that a two-factor model combining psychopathy and Machiavellianism, and keeping narcissism separate, has equivalent fit to the standard three factor model; as such, deciding between the optimal model may need to be based on theoretical grounds (12). However, a recent meta-analysis [102 studies, *N* = 46,234; (13)] suggests that the DT model inadequately captures the malevolent side of personality. Machiavellianism and psychopathy were more strongly linked to adverse psychosocial outcomes than Narcissism. Moreover, once psychopathy had been controlled, it alone remained significantly associated with all of the considered outcomes (including direct aggression). In comparison, the majority of the average effect sizes for both Machiavellianism and narcissism became considerably smaller and mostly non-significant (apart from interpersonal difficulties for both and antisocial tactics for Machiavellianism). These findings suggest the DT traits should be treated as independent constructs. Finally, examining the factorial structure of the DT traits, another recent study showed better model fit for a single latent dark core dimension compared to conceptualizing three independent traits (9), suggesting that the dark personalities are best represented through the single dark core and rendering the individual traits redundant. The mixed findings in the literature may partly be attributable to the measurement tools used, and their respective reliability and validity issues (8). Taken together, it is heavily debated whether the DT traits are best conceptualized as (i) three unique monads (single constructs), (ii) a Machiavellianism and psychopathy dark dyad, with narcissism as a separate construct, or (iii) a single joined dark triad core subsuming the three traits in predicting maladaptive behavior.

### Dark Traits and Different Forms of Aggression

Each DT trait has been linked to different forms of aggressive behaviors. Psychopathy is the strongest predictor of physical and premeditated aggression (13, 24–26); however, under certain conditions of response provocation, narcissistic and Machiavellian individuals are also likely to act aggressively. For example, narcissistic individuals are particularly responsive to ego-threat [i.e., any perceived attack on their self-image; (4)]. Whilst direct aggression has been extensively studied in the context of the DT, less is known about associations with indirect forms of aggression (i.e., those that are manipulative and more covert in nature). For example, whilst psychopathy has been linked to direct bullying behaviors in adults, narcissism and Machiavellianism have been linked to more indirect methods of intimidation (27). However, the association for narcissism was weak and potentially due to the shared variance with psychopathy (27), which would be in line with the findings of the recent meta-analysis (13).

Indirect relational aggression (IRA) reflects clandestine behaviors, aimed at damaging relationships and social status [e.g., peer group exclusion, rumor spreading, gossiping; (28)]. In this context, Machiavellianism has been linked to increased relational aggression in both adults and children, indicating that the cold, calculating nature suits a less direct and less physical use of force (29, 30). Similarly, whilst psychopathy and pathological vulnerable narcissism (more emotional, anxious and sensitive to criticism and rejection) have both been linked with increased reactive and proactive relational aggression in adults, grandiose narcissism (more akin to the DT-Narcissism) was linked to reduced relational aggression (31). Thus, in terms of both direct and indirect types of aggression more specific associations can be observed for the individual DT traits with different expressions of aggressive behavior.

### Empathy Deficits at the Core of Aggressive Behavior: Which Ones?

If the dark core is proposed to be underpinned by a lack of empathy that drives increased aggression in the DT, we would expect to see similar patterns across the DT. However, the unique associations of each DT trait with different expressions of aggressive behaviors are likely driven by more distinct affective and cognitive empathic deficits. Empathy is a multidimensional construct [e.g., (32)]—constituting cognitive and affective components. Cognitive empathy has been defined as the capacity to recognize and understand another person's mental states—*knowing what someone thinks*. Such perspective taking is essential for predicting behavior of others and manipulating them (33). Affective empathy involves a vicarious response to the emotional display of others—*feeling what someone feels*. It is this type of empathy that is implicated in the functioning of the Violence Inhibition Mechanism model [VIM; (6)], and the selective impairment in this capacity is thought to underpin aggression at least in psychopathy (7, 34). In addition to deficits in affective empathy, impairment in *affect-related* cognitive empathy–defined as the inability to *know* (rather than feel) *the emotions of another*—were also found in institutionalized offenders with psychopathic tendencies (35). By extension, if driven by the unempathic dark core, these mechanisms would then also apply to the other two traits. Indeed, affective empathy deficits have been linked to all three dark traits (individually and within the context of the DT), indicating a mutual inability to share the emotional experience of others (15, 36). However, once shared variance was accounted for, these findings were driven by psychopathy (15). On the other hand, there is also some evidence that all three DT traits are associated with reduced cognitive empathy, though this was driven only by psychopathy once shared variance was accounted for (37). In other cases, cognitive empathy appeared to be spared, or even increased in the case of Narcissism, which would suggest normal or better understanding of others' thoughts and intentions (15). Thus, the links between the DT and cognitive empathy deficits are less consistent.

Mixed findings might be due to the jingle-jangle fallacy, whereby different definitional constructs of cognitive (or affective) empathy have been measured and conflated across different studies (38). Moreover, whilst most studies have focused on general or more basic assessments of affective or cognitive empathy, a more fine-tuned facet approach within those types of empathic deficits may be more useful in predicting more specific forms of aggression (39). Reniers et al. (40) devised a multidimensional model of empathy distinguishing between different aspects of cognitive and affective empathy. Firstly, in their working definition cognitive empathy is the attribution of emotion (rather than cognition) more akin to the affect-related cognitive empathy construct above. Affective empathy is defined as sensitivity to and experience of others' feelings (rather than sharing or being aware of others' feelings, and distinct from sympathy). Their resulting model (and measurement instrument) encompasses perspective taking–*understanding and seeing others' emotional perspective*–and online simulation–*understanding another's perspective by imagining what they are feeling* as cognitive empathy components, whereas affective empathy comprises emotional contagion–*automatic mirroring of another's emotions*, proximal responsivity–*affective response to emotional cues of others*, and peripheral responsivity–*affective response to emotional cues in detached contexts (e.g., immersive settings like TV)*. With a few exceptions, (different factors of) psychopathy and Machiavellianism were associated with deficits across those scales and affective empathy was more strongly linked to expressive and instrumental forms of aggression; however, shared variance and mediation effects were not assessed in these analyses to derive a more informative distinct pattern of associations and predictions for the current study. Thus, the exact nature of such specific deficits in the DT traits remains unclear, as to the knowledge of the authors, prior research has not looked at these facets of cognitive and affective empathy in the context of the DT. Therefore, the current study examines these links controlling for the overlap of the DT traits to assess their unique and shared associations with those facets of affective and cognitive empathy in more detail. As such we want to examine which of these facets underpins the dark core (if supported) and which are more uniquely linked to individual traits.

Moreover, whilst direct forms of aggression have been examined within the VIM model, the extent to which this model applies to indirect aggression is less clear. Cognitive empathy has been shown to mediate the link between callous-unemotional (CU) and reactive relational aggression in women, indicating that women with reduced perspective taking skills are more likely to engage in relational aggression (41). Thus, whilst affective empathy has been proposed to play a central role in direct physical aggression [*cf*. VIM model; (6)], indirect aggression may be driven by cognitive empathy components. Given that distinct brain systems underpin those processes (42), it is clear that distinguishing those systems is crucial to further our understanding of the distinct motivational and behavioral mechanisms and pathways involved. Moreover, delineating those associations is important in terms of their theoretical implications as to whether such deficits are driven by an unempathic dark core and as such are common across all DT traits. This extends further to a practical need to better understand which subtypes of empathy drive different forms of aggression in order to inform more targeted intervention strategies.

### Summary and Hypotheses

There is a debate over the conceptualization of the DT traits as monad, dyad or triad, and whether the expression of distinct empathic deficits is linked to the unique or shared (aka dark CU core) variance of the three traits. Also little is known about the extent to which they subsequently promote similar or distinct patterns of indirect relational aggression. Few studies have taken a more fine-tuned facet approach to examine the unique and shared empathic deficits in the DT to examine whether they jointly or uniquely underpin aggressive behavior. Delineating these association will directly address one of the most prominent debates in the field and have strong theoretical implications in terms of the usefulness of the concept of the Dark Triad or indeed the individual dark triad traits.

The aim of this study was to examine which conceptualization of the DT best explains the relationships between the DT traits, empathy and indirect relational aggression: (1) an unique trait contribution of three *dark monads*, (2) a *dark dyad* (Machiavellianism and psychopathy) with a separate narcissism construct, or (3) a joint *dark triad* (Machiavellianism, psychopathy and narcissism). The study examined their unique and shared relations to distinct facets of cognitive and affective empathy, and different forms of IRA—social exclusion, malicious humor, and guilt induction. As with direct aggression, we hypothesized that, psychopathy will be the strongest predictor of all three IRA behaviors, whereas the manipulative and strategic nature of Machiavellianism will be linked to social exclusion and guilt induction. Both affective and cognitive empathy deficits were expected to be more (similar and) apparent in psychopathy and Machiavellianism. Specifically impairment in cognitive empathy components were expected to mediate the relationship of psychopathy and Machiavellianism with IRA. For narcissism, we did not expect empathy deficits or direct links to IRA after controlling for the other two traits.

## Methods

### Participants and Procedures

Three-hundred and one participants [262 females;^1^ age range 18–71 years, *M* = 26.87, *SD* = 11.66] were recruited from two UK University participant pools and via general online participation schemes. Ethical approval was obtained from the University Ethics Committees.

### Measures

#### The Dark Triad

Machiavellianism, narcissism, and psychopathy were measured using the 27-item Dark Triad of Personality Scale [D3-Short; (17)], scored on a 5-point Likert scale (1 = strongly disagree to 5 = strongly agree). Mean scores are calculated for the three subscales (9 items each) with higher scores indicating higher level of DT traits. The SD3 is a reliable measure with Cronbach's alphas ranging from 0.77 to 0.80, and respective associations with standard measures of psychopathy, Machiavellianism, and narcissism (13).

#### Empathy

Empathy was measured using the Questionnaire of Cognitive and Affective Empathy [QCAE; (40)] comprising 31 items in total scored on a 4-point Likert scale (1 = strongly agree to 4 = strongly disagree). Cognitive empathy consists of two facets: (1) perspective taking (PT; 10 items)–understanding internal mental states of others; and (2) online simulation (OS; 9 items)–understanding another's perspective by imagining what they are feeling. Affective empathy splits into three facets: (1) emotional contagion (EC; 4 items)–automatic copying of another's emotions, (2) proximal responsivity (ProR; 4 items)–response to emotional cues of others, and (3) peripheral responsivity (PerR; 4 items)–response to emotional cues in immersive settings. Cronbach's alphas range from 0.65 to 0.85. Scale scores were calculated by summing respective items.

#### Indirect Relational Aggression

Indirect relational aggression was measured using the Indirect Aggression Scale – Aggressor version [IAS-A; (28)]. The 25-item IAS-A consists of three subscales: Social Exclusion (SE; 10 items); Malicious Humor (MH; 9 items) and Guilt Induction (GI; 6 items). Participants indicate to what extent they had behaved aggressively during the last 12 months on a 5-point Likert scale (1 = never to 5 = regularly). Mean scores were obtained for each subscale. Cronbach's alphas range from 0.81 to 0.84.

### Statistical Analyses

Preliminary analyses revealed that several variables were skewed^2^; however, maximum likelihood estimators can provide estimates that are robust to non-normality (45). To gain a clearer understanding of how empathy relates to indirect aggression, the first step of analysis regressed all three IRA outcome variables on the five empathy variables. Following this, three main path models were specified from zero-order correlations and estimated using maximum likelihood in order to assess the fit of the different DT conceptualizations, and examine the relationships between the DT traits, empathy and indirect aggression factors. The first model assessed the unique contribution of the DT traits as *monads*, whereby the DT traits (Machiavellianism [M], psychopathy [P] and narcissism [N]) were separate observed variables and specified as correlated in order to account for their shared variance. The other two models assessed shared dark core contributions. The *dark dyad* model tested a latent variable with Machiavellianism and psychopathy as indicators, and narcissism as separate observed variable^3^, the *dark triad* model tested a latent variable with all three DT traits as indicator variables. Direct paths were specified from the DT traits compositions to IRA variables (with and without direct paths between Narcissism and IRA, and from the DT traits compositions to cognitive (PT and OS) and affective empathy variables (EC, ProR, PerR). Pathways were tested relating to the significant paths from the empathy variable/s (based on the regression results) to the IRA outcome variables (SE, MH, GI). Furthermore, empathy subscale scores were correlated with one another. Indirect effects were examined where applicable.

All analyses were undertaken in Muthén and Muthén (47) and if not stated otherwise, estimates reported are based on STDYX standardization. Model fit was deemed adequate with a non-significant chi-square value (taking sample size considerations into account), a root mean square error of approximation (RMSEA) below 0.05, a standardized root mean square residual (SRMR) below 1, and comparative fit (CFI) and Tucker-Lewis indices (TLI) above 0.90 (48).

## Results

### Descriptive Statistics

Mean, standard deviations and Cronbach's alphas for the DT traits, cognitive and affective facets of empathy and IRA are displayed in Table 1. All variables show good reliability with alphas ranging between 0.74 and 0.92.

**Table 1:** Descriptive statistics for all variables.

**Measures**	**Alpha**	**Mean**	***SD***
**DARK TRIAD**			
Machiavellianism	0.80	2.87	0.66
Psychopathy	0.75	2.06	0.59
Narcissism	0.74	2.65	0.60
**INDIRECT RELATIONAL AGGRESSION**			
Social exclusion	0.89	1.50	0.60
Malicious humor	0.89	1.45	0.60
Guilt induction	0.90	1.50	0.74
**EMPATHY**			
Perspective taking	0.92	32.80	5.30
Online simulation	0.87	27.50	4.90
Emotional contagion	0.85	11.00	8.80
Proximal responsivity	0.74	12.00	2.40
Peripheral responsivity	0.74	11.70	2.87

### Relationship of Empathy to IRA

Regressing all empathy variables on the three IRA outcome variables while controlling for age and sex led to a significant prediction of variability in SE (12.4%), MH (15.3%) and GI (14.9%) (all *p*s < 0.001). Looking at the predictor variables, only OS significantly and negatively predicted SE (unstandardized b = −0.042), MH (unstandardized b = −0.034), and GI (unstandardized b = −0.047) (all *p*s < 0.001). The standardized beta values can be seen in Table 2 (zero-order correlations can be found in the Supplementary Material). Consequently, the path model tests specified a path from the empathy variable OS only to all IRA variables.

**Table 2:** Standardized betas from the regression results of empathy predicting IRA.

	**SE**	**MH**	**GI**
Perspective taking	0.08	0.01	0.11
Online simulation	−0.34^***^	−0.28^***^	−0.31^***^
Emotional contagion	−0.07	−0.05	0.02
Proximal responsivity	0.03	−0.04	−0.11
Peripheral responsivity	−0.05	−0.06	−0.10
Sex	0.02	−0.09	0.05
Age	−0.03	−0.13^*^	−0.01

*** p < 0.001;

* *p < 0.05. SE, social exclusion; MH, malicious humor; GI, guilt induction*.

### Specified Path Models Testing the Conceptualization of DT Traits

**Figure 1:**
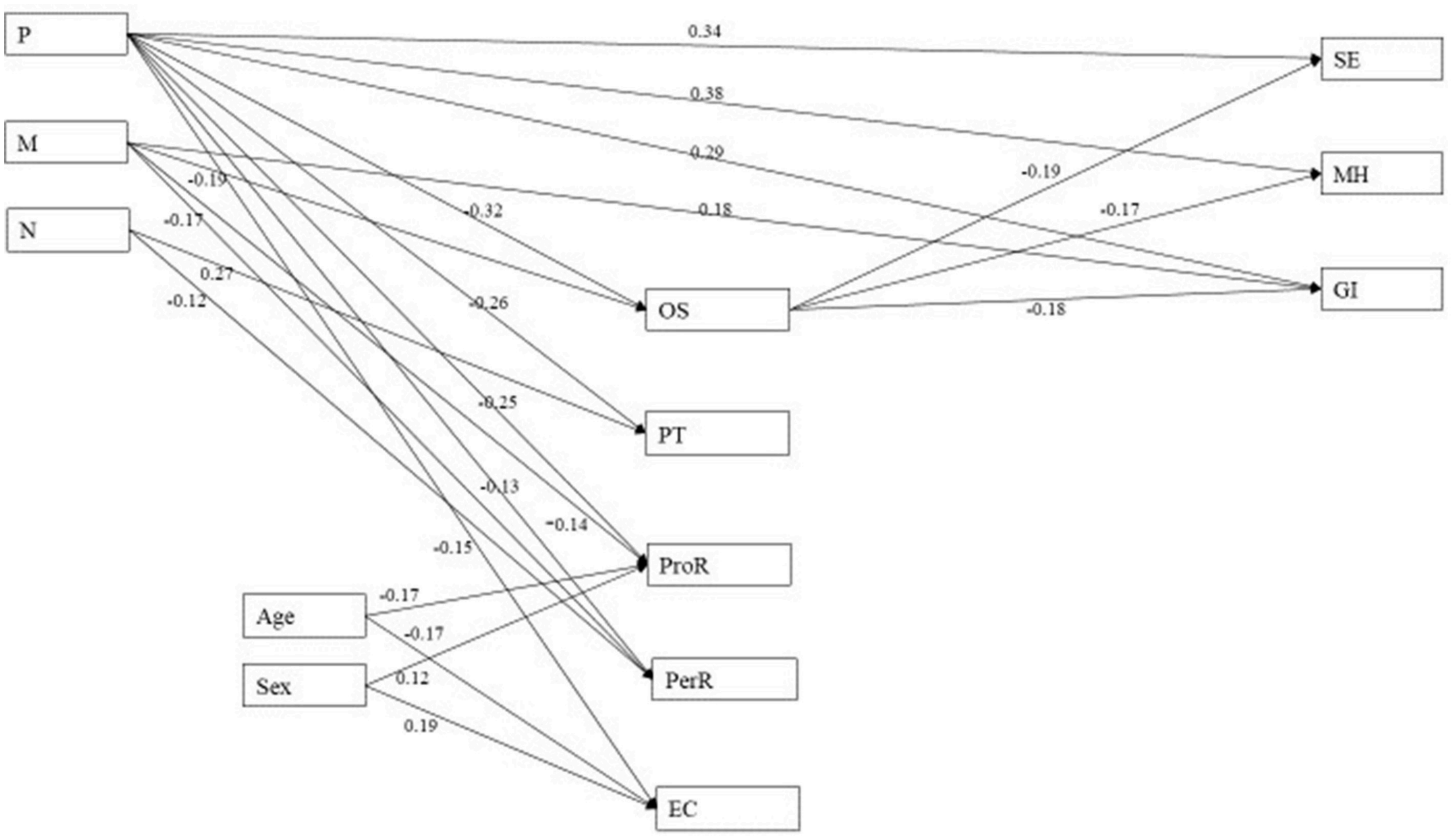
Path model of significant paths between DT, empathy and IRA. Values shown are STDYX estimates; paths significant at *p* < 0.05. P, psychopathy; M, Machiavellianism; N, narcissism; OS, online simulation; PT, perspective taking; ProR, proximal responsivity; PerR, peripheral responsivity; EC, emotional contagion; SE, social exclusion; MH, malicious humor; GI, guilt induction.

Model fit statistics are presented in Table 3. When direct paths from all DT traits to IRA were specified, Model 1 (*dark monads*) was not deemed a good fit: other than the SRMR, none of the indicators reached acceptable levels. Model 2 (*dark dyad*) also showed a poor fit, despite the SRMR and the CFI reaching acceptable limits. Model 3 (*dark triad*) showed a questionable fit: while CFI and SRMR reached acceptable thresholds, and TLI was only slightly below, RMSEA did not show adequate fit. These analyses confirmed that there were no significant paths between N and any of the IRA variables. Model 2a (*dark dyad* model without paths from N to IRA) did not reach acceptable model fit as both the RMSEA and the TLI missed specified cut-offs. However, Model 1a (*dark monads* model without paths between N and IRA) was deemed to fit as the Chi-square is non-significant, and all fit indices reach the cut-off values, even though the upper boundary of the 90% confidence interval of the RMSEA is not below 0.08 (49). This model also fit when age and sex were controlled for (model 1a_control), even though the upper boundary of the 90% confidence interval of the RMSEA was not below 0.08. A path model with standardized estimates and showing significant paths only is shown in Figure 1.

**Table 3:** Model fit statistics for all specified models.

**Model**	**Chi-square (df, p)**	**RMSEA (90% CI)**	**SRMR**	**CFI**	**TLI**
1. Monads (No-Core)	123.108 (4, *p* < 0.001)	0.315 (0.268–0.364)	0.076	0.879	−0.573
1a. Monads, no paths N to IRA	3.401 (3, *p* = 0.334)	0.021 (0.000–0.102) Probability RMSEA ≤ 0.05 0.616	0.014	1.000	0.993
1a_control. Monads, no paths N to IRA, age/sex controlled for	3.678 (3, *p* = 0.2984)	0.027 (0.000–0.105) Probability RMSEA < = 0.05 0.582	0.013	0.999	0.985
2. Dyad (Partial Core)	83.716 (9, *p* < 0.001)	0.166 (0.135–0.200)	0.087	0.934	0.596
2a. Dyad, no paths N to IRA	86.068 (12, *p* < 0.001)	0.143 (0.116–0.172)	0.095	0.934	0.699
3. Triad (Full Core)	50.614 (16, *p* < 0.001)	0.085 (0.059–0.112)	0.037	0.969	0.895

With regards to the empathy variables, only P was negatively related to all (*p*s ≤ 0.043), whereas M was negatively related to OS and PeriR only (*p*s ≤ 0.011). N was negatively related to PeriR (*p* = 0.037), as well as positively to PT (*p* < 0.001). In terms of the relationship with IRA, only P was positively related to all three outcomes (*p* < 0.001), M only significantly positively to GI (*p* = 0.004). Both M and P also negatively predicted OS (*p*s ≤ 0.001), suggesting OS as partial mediator. Furthermore, M may have indirect effects on SE and MH through OS, as OS is negatively related to all IRA outcome variables (*p*s ≤ 0.001). The analysis controlled for age and sex which were only significantly related to the ProR and EC empathy variables (*p*s < 0.05), and not significantly related to any of the IRA variables.

## Discussion

There has been considerable debate in the literature on the conceptualization of the dark traits as individual traits or sharing a dark core, with impaired empathy at the center [e.g., (9–13)]. In this study, we examined the different conceptualizations of the DT traits–as *dark monads* (three single units), a *dark dyad* of Machiavellianism and psychopathy (with narcissism kept separate), and a *dark triad* with a shared core as a latent variable–in their distinct and joint associations with cognitive and affective empathy facets and the link to indirect relational aggression. All three dark traits were associated with reduced peripheral responsivity, suggesting this facet of affective empathy may underpin the shared dark core; however, peripheral responsivity was unrelated to IRA, challenging the notion that the unempathic core of the DT drives relational aggression. Importantly, the monads model without links between narcissism and IRA showed the best fit, whereas the joint dark core models (with or without narcissism) showed unsatisfactory fits to our data. Our findings show that, individually, psychopathy is the most severe trait in terms of its global maladaptive relationships with empathy and IRA, and Machiavellianism produces weaker but more specific deficits, while narcissism is spared. This suggests that the DT traits are best viewed as three independent personality traits, rather than a joint (latent) dyad or triad core, at least in the prediction of these specific empathic deficits and indirect relational aggression.

### The Distinct Profiles of the DT Traits

The current findings support distinct profiles of the individual DT traits due to their specific characteristics that drive distinct expressions of empathic deficits (and spared capacities) and indirect aggressive behavior. Psychopathy was compromised on all levels of cognitive and affective empathy, and all types of relational aggression. Thus, consistent with previous research on direct aggression (4, 13), psychopathy showed a more severe and global pattern of maladaptive outcomes. Machiavellianism and narcissism showed more specific links to empathy. Only a partial cognitive deficit–online simulation–was observed in Machiavellianism, whereas perspective taking appears intact; being able to predict another's intentions would facilitate their manipulation strategies (33). Machiavellianism predicted only guilt induction reflecting their use of manipulation (e.g., emotional blackmail) to influence others (28). Narcissism, on the other hand, was not related to any IRA, and indeed model fit was only achieved when the paths between narcissism and IRA were removed. This questions to what extent previously observed narcissism-aggression links [e.g., (27)] were driven by the shared variance with psychopathy and/or Machiavellianism. Moreover, narcissism was linked to increased perspective taking ability, perhaps enabling them to create a more favorable self-image through understanding and predicting others reactions. Thus, the spared and more adaptive associations of narcissism, once psychopathy and Machiavellianism are controlled for, emphasize the importance of teasing DT traits apart to examine their unique variance.

### What Empathy Deficits Underpin the Dark Core and Which Drive Aggression in the DT: Implications for the VIM

This study examined whether by extension, the same mechanisms of the Violence Inhibition Mechanism model [VIM; (6, 34)] apply to (i) Machiavellianism and narcissism – if driven by the shared dark core, and/or (ii) other expressions of aggressive behavior such as relational aggression. Studying relational aggression in this context, means less observable, more indirect means of antagonistic behaviors in the Dark Triad traits can be explored, and allows us to test theoretical assumptions in different contexts. Our monad model conceptualizations of the dark traits as independent constructs and without an unempathic core that drives IRA challenge both assumptions.

Firstly, all three DT traits shared at least one affective empathy deficit—reduced peripheral responsivity, which arguably may underlie their shared dark core and reflect selfishness and tough-mindedness, and to care less about others in detached contexts. However, this shared deficit in peripheral responsivity did not drive indirect relational aggression. This does not necessarily contradict the extension of the VIM to the dark core as this shared deficit may still drive physical aggression; however, it does not apply to less direct means of aggression studied here.

Secondly, only the *affect-related cognitive* empathy facet online simulation was a partial mediator, driving indirect effects on IRA for Machiavellianism and psychopathy. Indeed, relational aggression has previously been associated with cognitive empathy deficits in women (41), and the current study supports this finding in a mixed sex sample. Though the current sample comprises an over-representation of the female sex, when controlled for, the main model fit was not affected and sex was not significantly related to any IRA variable suggesting that these mechanisms hold for males also. However, replication in a (predominantly) male sample is needed before drawing any firm conclusions. Though traditionally a lack of a*ffective* empathy has been seen as the hallmark deficit of psychopathy [e.g., VIM model; (6)], similar *affect-related cognition* deficits as found in the current study have been previously reported in criminal male offenders with psychopathic tendencies (35). Our findings do not contradict the classic VIM notion, however, they do highlight an additional nuance of its functioning in driving this specific type of aggression. Whilst a cognitive component of understanding intentions and cognitions of others is essential for manipulating and deceiving (33), a lack of understanding what individuals feel in response to that may indeed facilitate such behavior, and hence, drive indirect relational aggression as measured in the current study.

Our interpretations and comparisons with findings across the literature must be taken with care. Firstly, they are based on self-report measures rather than psychophysiological indices of empathic responses or emotional reactivity and actual observed aggressive behavior (e.g., in the lab). For example, future research should examine empathic deficits underpinning direct and indirect behavioral aggression using carefully designed behavioral and/or psychophysiological experimental paradigms that tap into these specific aspect. From a psychometric perspective, previous studies have used different measures of empathy, and none used the facet approach distinguishing between different aspects of affective and cognitive empathy in the context of the whole DT. As such, the jingle-jangle fallacy posed within empathy research (38) needs to be considered here. For example, both of Renier's et al (40) cognitive empathy scales, online simulation and perspective taking, are defined by the capacity to make attributions about affect–akin to *affect-related* cognitive empathy (35). As such, Renier's et al. (40) and Davis' (32) perspective taking scales present us with a jingle issue (inference that two measures with the same labels measure the same things). Whilst former is proposed to measures attributions about affective processing (e.g., *I can tell when someone is masking their true emotions; good at predicting how someone will feel*), latter is defined as the tendency to spontaneously adopt the psychological point of view of others and appears to take a more traditional conceptualization of perspective taking as attributions about others cognitions and mental states for most items [e.g., *see things from the “other guy's” point of view; look at everybody's side of a disagreement; imagining how things look from their perspective;* (32)]. Thus, they are not likely measuring the same type of empathic attributions. To complicate matters more, Reniers et al.' online simulation scale presents us with a jangle problem (inference that two measures with different labels measure different things) as the majority of its items (five out of nine) are actually from Davis' perspective taking scale. On face validity, four items clearly concern attributions of cognitions (as mentioned above), two tap into attributions of affect (e.g., *I try to imagine how I would feel if I was in their place*) and the rest could be interpreted as both (e.g., *I find it easy to put myself in somebody else's shoes*). Thus, this scale appears to not just encompass “an effortful attempt to put oneself in another person's position by imagining what that person is feeling” [(40); p. 90] but also how they may think and react (i.e., mental states). In other words, it is the capacity to simulate another's thoughts *and* emotions. Subsequently, the current findings suggest that indirect relational aggression in psychopathy and Machiavellianism is in fact underpinned by the impairment in the capacity to simulate both, other's cognitions and emotions.

### One Good, One Bad, One Ugly–Narcissism as the Odd One Out?

As IRA is more proactive—premeditated and instrumental—in nature, it would appeal more to strategic, pragmatic and manipulative individuals akin to Machiavellianism and psychopathy, rather than narcissism. In contrast, narcissism has been more associated with reactive aggression in response to perceived provocation [e.g., ego-threat; (17)]. Thus, our findings do not mean that narcissistic individuals are not aggressive *per se*, but suggest that IRA is a type of aggression they are unlikely to engage in—perhaps because it would not provide them with admiration and positive social standing they desire. Moreover, when studied independently, narcissism is split into two subtypes, grandiose and vulnerable (50), which manifest differently on general personality measures [e.g., the Big Five; (10)] and show diverging associations with hostility [e.g., vulnerable narcissism is linked to hostile behaviors once grandiosity and attention-seeking are controlled for; (51)]. However, DT measures such as the SD3 focus on grandiose narcissism at the expense of vulnerable narcissism. To thoroughly understand the relationship between narcissism and aggression, we need to consider the conceptualization of narcissism as utilized by various psychometric measures.

Moreover, research has considered Machiavellianism simply a less severe expression of clinical psychopathy (52). However, an overlap between constructs also reflects limited theoretical distinction, and suggests a need to refine measures of Machiavellianism, free from psychopathic traits (12). A recent meta-analysis revealed different degrees of overlaps for different DT measures: the SD3 incorporates more components of psychopathy, whereas the Dirty Dozen (53) shows more overlap with Machiavellianism and narcissism (13). Therefore, current findings need to be replicated with other measures of the DT traits as well as more comprehensive measures of each individual traits before drawing firm conclusions.

Though there appear adaptive qualities in narcissism, including self-confidence (54), their desire for social desirability leads to a tendency to exaggerate their abilities and reflect themselves in a positive light. This is a limitation when using self-reported measures to study individuals prone to dishonesty and may have affected our results. However, recent research suggests that even in large groups of psychopathic offenders response distortion was not a concern (55). Nevertheless, future studies may consider including measures of social desirability or lie-scales to control for such unwanted effects.

Finally, whilst our monads model without links between narcissism and IRA showed the best fit, recent bi-factorial modeling approaches suggest that the common core of dark traits might not depend on the individual DT traits but can be seen as a separate global construct (56, 57). Due to sample size restrictions we could not evaluate such models in the current study. It is important to note for the interpretation of our current best-fitting monad model, that we have partialled out the overlap between the DT traits to look at their unique impact. However, this can come under scrutiny because there is an interpretative difference between the concept of narcissism (measured as a whole) and the statistical variance that is left once the other traits have been accounted for (58). Thus, further research needs to focus on specifying what the differentiation of the whole and the partialled construct of Narcissism actually is (same applies for the other two traits).

### Conclusion

The current paper addressed one of the most debated questions as to whether the dark traits are best conceptualized as individual traits (monads) or shared constructs (dyad or triad)–with a joint dark core. In addition, we examined whether the joint dark core is underpinned by empathy, and whether this drives their aggression—a debate fuelled by a lack of empirical findings that distinguish facets of empathy underpinning the core and those more uniquely linked to the individual dark traits. Our findings suggest the DT traits are best conceptualized as distinct monads with unique pathways in terms of affective and cognitive empathy deficits and capacities, and their role in relational aggression. Narcissism showed the greatest differentiation from the other two, and a more adaptive nature with increased empathic capacities (perspective taking) and lack of association with IRA. This supports the notion that the DT traits are not equal members of the DT, and as such highlight the important distinction between them. Machiavellianism demonstrates more similar, though less global empathic deficits and relational aggression as psychopathy—as such adding further weight to research indicating greater overlap amongst these two traits. Importantly, in contrast to theoretical models focusing on affective empathy deficits in direct physical aggression, cognitive empathy deficits related to affect (and cognition) inference drive indirect relational aggression in psychopathy and Machiavellianism. Thus, our results highlight the importance of assessing empathy as a multidimensional construct in relation to DT traits and maladaptive outcomes, such as different forms of aggression.

Few studies have taken a more fine-tuned facet approach to examine the unique and shared empathic deficits in the DT and their link to aggressive behavior. Delineating the specific aspects involved is crucial to further our understanding of the distinct motivational and behavioral mechanisms involved, inform theoretical models of aggression in the DT such as the VIM, and more targeted intervention strategies for different expressions of aggressive behavior in the dark traits. However, in taking this approach studies need to carefully take the empathy jingle-jangle fallacy into account whereby inconsistent findings may be based on conceptual misconceptions about what is being measured, and therefore, care needs to be taken in the interpretation of findings and comparison across studies. Finally, the current findings only suggest that indirect relational aggression in non-clinical populations is partially mediated by reduced online simulation—whether this extends to direct physical aggression and forensic populations remains to be tested. Nevertheless, they are the first to show these unique pathways for the dark trait traits their links to specific empathic deficits and indirect relational aggression.

## Author Contributions

NH, AS, and CB conceptualized the initial project idea and designed the study with further contributions from VE. JF contributed to the implementation and acquisition of the data. NH, FK, and JF contributed to the data analyses and all authors to the interpretation of findings. NH and JF wrote the initial draft of the paper and subsequent revisions. All authors were involved in editing the individual drafts. NH revised the final draft for submission to the special issue with final editing support by AS. All authors approve publication of the content and agree to be accountable for all aspects of the work.

### Conflict of Interest Statement

The authors declare that the research was conducted in the absence of any commercial or financial relationships that could be construed as a potential conflict of interest.

## Footnotes

^1^Participants were asked to indicate their biological sex with males, females and other as response options (none indicated latter); hence, we use sex rather than gender when we refer directly to our study data. When we refer to the wider literature, we are either concordant with what the respective authors use in their terminology or use both “sex/gender” terms [see (43)] if used interchangeably within the respective literature. Some previous research suggests that relational aggression is more prominent in women; however, a large meta-analysis shows minimal gender differences (44). Nevertheless, we control for sex in our analyses to account for the overrepresentation of females in the sample.

^2^Psychopathy was positively (*z* = 5.67), PT, OS, ProR, and PerR were negatively skewed (*z* scores between −3.59 and −5.93). The IRA subscales showed the greatest skew (all *z* > 15).

^3^Models with two indicators for a latent variable are deemed problematic as construct representation is less accurate; however, they are nevertheless often specified in empirical research as this bias can be reduced when there is high communality between the indicators (46).

## References

[1] Preckel K Kanske P Singer T On the interaction of social affect and cognition: empathy, compassion and theory of mind. Curr Opin Behav Sci. (2018) 19:1–6. 10.1016/j.cobeha.2017.07.010

[2] Paulhus DL Williams KM The dark triad of personality: narcissism, machiavellianism, and psychopathy. J Res Personal. (2002) 36:556–63. 10.1016/S0092-6566(02)00505-6

[3] Jones DN Figueredo AJ The core of darkness: uncovering the heart of the dark triad. Eur J Personal. (2013) 27:521–31. 10.1002/per.1893

[4] Jones DN Paulhus DL Different provocations trigger aggression in narcissists and psychopaths. Soc Psychol Pers Sci. (2010) 1:12–8. 10.1177/194855060934759

[5] Blair RJR. Fine cuts of empathy and the amygdala: dissociable deficits in psychopathy and autism. Q J Exp Psychol. (2008) 61:157–70. 10.1080/17470210701508855 18038346

[6] Blair RJR A cognitive developmental approach to morality: investigating the psychopath. Cognition. (1995) 57:1–29. 10.1016/0010-0277(95)00676-P 7587017

[7] Blair RJR Mitchell DGV Peschardt KS Colledge E Leonard RA Shine JH Reduced sensitivity to others' fearful expressions in psychopathic individuals. Person Individ Differ. (2004) 37:1111–22. 10.1016/j.paid.2003.10.008

[8] Furnham A Richards SC Paulhus DL The dark triad of personality: a 10 year review. Soc Personal Psychol Comp. (2013) 7:199–216. 10.1111/spc3.12018

[9] Bertl B Pietschnig J Tran US Stieger S Voracek M More or less than the sum of its parts? Mapping the dark triad of personality onto a single dark core. Personal Individ Differ. (2017) 114:140–4. 10.1016/j.paid.2017.04.002

[10] Egan V Chan S Shorter GW The Dark Triad, happiness and subjective well-being. Personal Individ Differ. (2014) 67:17–22. 10.1016/j.paid.2014.01.004

[11] Egan V Hughes N Palmer E Moral disengagement, the dark triad and unethical consumer behavior. Personal Individ Differ. (2015) 76:123–8. 10.1016/j.paid.2014.11.054.

[12] Miller JD Hyatt CS Maples-Keller JL Carter NT Lynam DR. Psychopathy and machiavellianism: a distinction without a difference? J Personal. (2016) 85:439–53. 10.1111/jopy.12251 26971566

[13] Muris P Merckelbach H Otgaar H Meijer E The malevolent side of human nature: A meta-analysis and critical review of the literature on the dark triad (narcissism, machiavellianism, and psychopathy). Perspect Psychol Sci. (2017) 12:183–204. 10.1177/1745691616666070 28346115

[14] Rauthmann JF Acquisitive or protective self-presentation of dark personalities? Associations among the dark triad and self-monitoring. Personal Individ Differ. (2011) 51:502–8. 10.1016/j.paid.2011.05.008

[15] Wai M Tiliopoulos N The affective and cognitive empathic nature of the dark triad of personality. Personal Individ Differ. (2012) 52:794–9. 10.1016/j.paid.2012.01.008

[16] Hare RD Neumann CS. Psychopathy as a clinical and empirical construct. Ann Rev Clin Psychol. (2008) 4:217–46. 10.1146/annurev.clinpsy.3.022806.091452 18370617

[17] Jones DN Paulhus DL. Introducing the short dark triad (SD3) a brief measure of dark personality traits. Assessment. (2014) 21:28–41. 10.1177/1073191113514105 24322012

[18] Vernon PA Villani VC Vickers LC Harris JA A behavioral genetic investigation of the dark triad and the big 5. Personal Individ Differ. (2008) 44:445–52. 10.1016/j.paid.2007.09.007

[19] O'Boyle JEH Forsyth DR Banks GC McDaniel MA. A meta-analysis of the dark triad and work behavior: a social exchange perspective. J Appl Psychol. (2012) 97:557–79. 10.1037/a0025679 22023075

[20] Book A Visser BA Volk AA Unpacking “evil”: claiming the core of the dark triad. Personal Individ Differ. (2015) 73:29–38. 10.1016/j.paid.2014.09.016

[21] Lee K Ashton MC Psychopathy, Machiavellianism, and narcissism in the Five-Factor Model and the HEXACO model of personality structure. Pers Indiv Differ. (2005) 38:1571–82. 10.1016/j.paid.2004.09.016

[22] Jonason PK Lyons M Bethell EJ Ross R Different routes to limited empathy in the sexes: examining the links between the Dark Triad and empathy. Pers Indiv Differ. (2013) 54:572–6. 10.1016/j.paid.2012.11.009

[23] Kajonius PJ Persson BN Rosenberg P Garcia D. The (mis)measurement of the dark triad dirty dozen: exploitation at the core of the scale. PeerJ. (2016) 1:e1748. 10.7717/peerj.1748.eCollection2016. 26966673 PMC4782707

[24] Jones DN Neria AL The Dark Triad and dispositional aggression. Personal Individ Differ. (2015) 86:360–4. 10.1016/j.paid.2015.06.021

[25] Lämmle L Oedl C Ziegler M Don't threaten me and my dark side or even self-harm won't stop me from hurting you. Personal Individ Differ. (2014) 67:87–91. 10.1016/j.paid.2013.12.024

[26] Paulhus DL Curtis SR Jones DN. Aggression as a trait: the Dark Tetrad alternative. Curr Opin Psychol. (2018) 19:88–92. 10.1016/j.copsyc.2017.04.007 29279229

[27] Baughman HM Dearing S Giammarco E Vernon PA Relationships between bullying behaviours and the dark triad: a study with adults. Personal Individ Differ. (2012) 52:571–5. 10.1016/j.paid.2011.11.020

[28] Forrest S Eatough V Shevlin M Measuring adult indirect aggression: The development and psychometric assessment of the indirect aggression scales. Aggress Behav. (2005) 31:84–97. 10.1002/ab.20074

[29] Abell L Brewer G Machiavellianism, self-monitoring, self-promotion and relational aggression on Facebook. Comput Hum Behav. (2014) 36:258–62. 10.1016/j.chb.2014.03.076

[30] Kerig PK Stellwagen KK Roles of callous-unemotional traits, narcissism, and Machiavellianism in childhood aggression. J Psychopathol Behav Assess. (2010) 32:343–52. 10.1007/s10862-009-9168-7

[31] Knight NM Dahlen ER Yowell EB Madson MB The HEXACO model of personality and dark triad in relational aggression. Personal Individ Differ. (2018) 122:109–14. 10.1016/j.paid.2017.10.016

[32] Davis MH Measuring individual differences in empathy: Evidence for a multidimensional approach. J Personal Soc Psychology. (1983) 44:113–26.

[33] Smith A Cognitive empathy and emotional empathy in human behavior and evolution. Psychol Record. (2010) 56:1 10.1007/BF03395534

[34] Blair RJR. Responding to the emotions of others: dissociating forms of empathy through the study of typical and psychiatric populations. Conscious Cogn. (2005) 14:698–718. 10.1016/j.concog.2005.06.004 16157488

[35] Shamay-Tsoory SG Harari H Aharon-Peretz J Levkovitz Y. The role of the orbitofrontal cortex in affective theory of mind deficits in criminal offenders with psychopathic tendencies. Cortex. (2010) 46:668–77. 10.1016/j.cortex.2009.04.008 19501818

[36] Ali F Amorim IS Chamorro-Premuzic T Empathy deficits and trait emotional intelligence in psychopathy and Machiavellianism. Personal Individ Differ. (2009) 47:758–62. 10.1016/j.paid.2009.06.016.

[37] Jonason PK Krause L The emotional deficits associated with the dark triad traits: cognitive empathy, affective empathy, and alexithymia. Personal Individ Differ. (2013) 55:532–7. 10.1016/j.paid.2013.04.027

[38] Hall AJ Schwartz R. Empathy present and future. J Soc Psychol. (2018). 10.1080/00224545.2018.1477442. [Epub ahead of print]. 29781776

[39] Heym N Fergusson E Lawrence C The P-psychopathy continuum: facets of Psychoticism and their associations with psychopathic tendencies. Personal Individ Differ. (2013) 54:773–8. 10.1016/j.paid.2012.12.001

[40] Reniers RL Corcoran R Drake R Shryane NM Völlm BA. The QCAE: a questionnaire of cognitive and affective empathy. J Personal Assess. (2011) 93:84–95. 10.1080/00223891.2010.528484 21184334

[41] White BA Gordon H Guerra RC Callous–unemotional traits and empathy in proactive and reactive relational aggression in young women. Personal Individ Differ. (2015) 75:185–9. 10.1016/j.paid.2014.11.031

[42] Shamay-Tsoory SG Aharon-Peretz J Perry D. Two systems for empathy: a double dissociation between emotional and cognitive empathy in inferior frontal gyrus versus ventromedial prefrontal lesions. Brain. (2009) 132:617–27. 10.1093/brain/awn279 18971202

[43] Rippon G Jordan-Young R Kaiser A Fine C. Recommendations for sex/gender neuroimaging research: key principles and implications for research design, analysis, and interpretation. Front Hum Neurosci. (2014) 8:650. 10.3389/fnhum.2014.00650 25221493 PMC4147717

[44] Card NA Stucky BD Sawalani GM Little TD. Direct and indirect aggression during childhood and adolescence: a meta-analytic review of gender differences, intercorrelations, and relations to maladjustment. Child Dev. (2008) 79:1185–229. 10.1111/j.1467-8624.2008.01184.x 18826521

[45] Du Toit S Du Toit M Joreskog KG Sorbom D Interactive LISREL: User's Guide. Chicago, IL: Scientific Software International (1999).

[46] Little TD Lindenberger U Nesselroade JR On selecting indicators for multivariate measurement and modeling with latent variables: when “good” indicators are bad and “bad” indicators are good. Psychol Methods. (1999) 4:192 10.1037/1082-989X.4.2.192

[47] Muthén LK Muthén BO Mplus 7.3. Muthén & Muthén (2014).

[48] Hu LT Bentler PM Cutoff criteria for fit indexes in covariance structure analysis: Conventional criteria versus new alternatives. Struct Equ Model Multidisciplin J. (1999) 6:1–55. 10.1080/10705519909540118

[49] MacCallum RC Browne MW Sugawara HM Power analysis and determination of sample size for covariance structure modeling. Psychol Methods. (1996) 1:130–49. 10.1037/1082-989X.1.2.130

[50] Pincus AL Lukowitsky MR. Pathological narcissism and narcissistic personality disorder. Ann Rev Clin Psychol. (2010) 6:421–46. 10.1146/annurev.clinpsy.121208.131215 20001728

[51] Miller JD Hoffman BJ Gaughan ET Gentile B Maples J Keith Campbell W. Grandiose and vulnerable narcissism: a nomological network analysis. J Personal. (2011) 79:1013–42. 10.1111/j.1467-6494.2010.00711.x 21204843

[52] Glenn AL Sellbom M. Theoretical and empirical concerns regarding the dark triad as a construct. J Personal Disord. (2015) 29:360–77. 10.1521/pedi_2014_28_162 25248015

[53] Jonason PK Webster GD. The dirty dozen: a concise measure of the dark triad. Psychol Assess. (2010) 22:420. 10.1037/a0019265 20528068

[54] Rauthmann JF Kolar GP How “dark” are the Dark Triad traits? Examining the perceived darkness of narcissism, Machiavellianism, and psychopathy. Personal Individ Differ. (2012) 53:884–9. 10.1016/j.paid.2012.06.020

[55] Watts AL Lilienfeld SO Edens JF Douglas KS Skeem JL Verschuere B. Does response distortion statistically affect the relations between self-report psychopathy measures and external criteria? Psychol Assess. (2016) 28:294. 10.1037/pas0000168.supp 26121383

[56] McLarnon MJ Tarraf RC. The dark triad: specific or general sources of variance? A bifactor exploratory structural equation modeling approach. Personal Individ Differ. (2017) 112:67–73. 10.1016/j.paid.2017.02.04919141049

[57] Moshagen M Hilbig BE Zettler I. The dark core of personality. Psychol Rev. (2018) 125:656–88. 10.1037/rev0000111 29999338

[58] Vize CE Lynam DR Collison KL Miller JD. Differences among dark triad components: a meta-analytic investigation. Personal Disord Theor Res Treat. (2018) 9:101–11. 10.1037/per0000222 27736106

