# Supplementary material for: Empathy at the Heart of Darkness: Empathy Deficits That Bind the Dark Triad and Those That Mediate Indirect Relational Aggression
Source: Front Psychiatry. 2019 Mar 12;10:95. doi: 10.3389/fpsyt.2019.00095 (PMC6423894; doi:10.3389/fpsyt.2019.00095)
Supplement: Supplementary file 1 [file Table_1.docx]

Supplementary Table: Zero-order correlations between all variables

|  | Sex | Age | DT Machiavelianism | DT Narcissism | DT Psychopathy | Perspective Taking | Online Simulation | Emotional Contagion | Proximal Resp | Peripheral Resp | Social Exclusion | Malicious Humour |
| --- | --- | --- | --- | --- | --- | --- | --- | --- | --- | --- | --- | --- |
| Age | -.050 | 1 |  |  |  |  |  |  |  |  |  |  |
| DT Machiavellianism | -.044 | -.200^**^ | 1 |  |  |  |  |  |  |  |  |  |
| DT Narcissism | -.091 | -.133^*^ | .369^**^ | 1 |  |  |  |  |  |  |  |  |
| DT Psychopathy | -.132^*^ | -.137^*^ | .495^**^ | .385^**^ | 1 |  |  |  |  |  |  |  |
| Perspective taking | .064 | .035 | .030 | .184^**^ | -.137^*^ | 1 |  |  |  |  |  |  |
| Online simulation | -.004 | .112 | -.324^**^ | -.106 | -.385^**^ | .475^**^ | 1 |  |  |  |  |  |
| Emotional contagion | .222^**^ | -.149^**^ | -.070 | -.117^*^ | -.175^**^ | .145^*^ | .319^**^ | 1 |  |  |  |  |
| Proximal responsivity | .167^**^ | -.117^*^ | -.229^**^ | -.125^*^ | -.307^**^ | .190^**^ | .465^**^ | .612^**^ | 1 |  |  |  |
| Peripheral responsivity | .099 | .009 | -.272^**^ | -.235^**^ | -.259^**^ | .148^*^ | .320^**^ | .307^**^ | .431^**^ | 1 |  |  |
| Social exclusion | .013 | -.054 | .272^**^ | .242^**^ | .426^**^ | -.095 | -.335^**^ | -.159^**^ | -.177^**^ | -.159^**^ | 1 |  |
| Malicious humour | -.105 | -.143^*^ | .319^**^ | .263^**^ | .493^**^ | -.159^**^ | -.346^**^ | -.183^**^ | -.227^**^ | -.189^**^ | .709^**^ | 1 |
| Guilt induction | .034 | -.035 | .369^**^ | .251^**^ | .430^**^ | -.072 | -.341^**^ | -.149^**^ | -.258^**^ | -.223^**^ | .655^**^ | .645^**^ |

* Correlation is significant at the 0.05 level (2-tailed).

** Correlation is significant at the 0.01 level (2-tailed).
